# PP-JPEG: A Privacy-Preserving JPEG Image-Tampering Localization

**DOI:** 10.3390/jimaging9090172

**Published:** 2023-08-27

**Authors:** Riyanka Jena, Priyanka Singh, Manoranjan Mohanty

**Affiliations:** 1 Research Group for Security and Privacy, Dhirubhai Ambani Institute of Information and Communication Technology, Gandhinagar 382004, India; 201921012@daiict.ac.in; 2 School of Electrical Engineering and Computer Science, The University of Queensland, Brisbane 4072, Australia; 3 School of Mathematical and Physical Science, University of Technology Sydney, Ultimo 2007, Australia; Manoranjan.Mohanty@uts.edu.au

**Keywords:** image forensics, tampering localization, copy-move forgery, Paillier encryption

## Abstract

The widespread availability of digital image-processing software has given rise to various forms of image manipulation and forgery, which can pose a significant challenge in different fields, such as law enforcement, journalism, etc. It can also lead to privacy concerns. We are proposing that a privacy-preserving framework to encrypt images before processing them is vital to maintain the privacy and confidentiality of sensitive images, especially those used for the purpose of investigation. To address these challenges, we propose a novel solution that detects image forgeries while preserving the privacy of the images. Our method proposes a privacy-preserving framework that encrypts the images before processing them, making it difficult for unauthorized individuals to access them. The proposed method utilizes a compression quality analysis in the encrypted domain to detect the presence of forgeries in images by determining if the forged portion (dummy image) has a compression quality different from that of the original image (featured image) in the encrypted domain. This approach effectively localizes the tampered portions of the image, even for small pixel blocks of size 10×10 in the encrypted domain. Furthermore, the method identifies the featured image’s JPEG quality using the first minima in the energy graph.

## 1. Introduction

The advancement of digital image-processing software has made it effortless to create manipulated images without any noticeable signs [1]. Consequently, people are losing faith in the trustworthiness and authenticity of digital images. This erosion of trust in digital imagery has far-reaching implications, impacting domains such as journalism, forensics, and legal proceedings. Hence, developing technologies to determine whether an image is altered is important.

Joint Photographic Experts Group (JPEG) [2] is the most widely used image format. Human eyes are less sensitive to high-frequency signals [3]. JPEG compression compresses the high-frequency information without losing the image quality. The tampered region has a different JPEG compression than the authentic region in a tampered JPEG image. Thus, it is difficult for the human eye to identify tampered digital images. However, such images usually have hidden clues and statistical artifacts [4]. Existing JPEG digital forensic technologies use these clues or artifacts to detect the tampering of images [5].

In this work, we investigate the following research question:

- RQ1: In the encrypted domain, how do we detect tampering in JPEG images when a low-quality JPEG image is inserted into a higher-quality JPEG image and vice versa?

The main objective is to develop a method that can effectively identify such forgeries while considering the practical applications in forensics investigations, where preserving the utility and privacy of user data is crucial. This approach is particularly relevant in real-world scenarios, where image tampering may occur for various malicious purposes. By detecting and locating forgeries, our method aids forensic investigations while minimizing the risk of compromising the privacy and utility of the original image data. We use forgery and tampering interchangeably throughout this paper.

This paper suggests a way to detect image forgery in a passive method [6]. We present a technique for detecting tampering in low-quality and high-quality images in the encrypted domain. This approach detects tampering when a low-quality JPEG image is placed into a higher-quality JPEG image and vice versa. It can come from combining two separate photographs of people into a single composite image, for example, or by splicing one person’s head onto another person’s body. In this method, part of the image is explicitly determined to be compressed at a quality different from the rest of the image. The method relies on examining the differences in the JPEG qualities between the forged part and the rest of the image in the encrypted domain. The original image is named the “featured” image, while the manipulated portion is named the “dummy” image. The technique involves identifying and locating the forged portion by saving the manipulated image multiple times with different image qualities and finding the range of JPEG qualities that best detect the forgery. Through experimentation involving various combinations of dummy and featured image qualities, the obtained results are analyzed to ascertain the most effective techniques for localizing image forgery in an encrypted domain.

We propose a novel approach for passive image forgery detection and explore resaving at different image qualities to improve forgery localization. The key contributions of the paper are as follows:

- The proposed method is evaluated using various scenarios of copy–move forgeries in the encrypted domain. Copy–move forgeries involve duplicating and pasting a portion of an image onto another part of the same image. During the testing, various combinations of images were used, including high-quality dummy images with low-quality featured images, low-quality dummy images with high-quality featured images, and equal JPEG quality for both dummy and featured images. The pixel block size of the manipulation area ranged from 50×50 to as small as 10×10. The test results showed that the proposed method successfully identified the tampered parts of the image in the encrypted domain. The technique worked well in finding manipulated areas, regardless of the image quality and block size variations. These findings suggest that the proposed method is useful for detecting and locating copy–move forgeries in images in an encrypted domain.
- The forgery detection results were analyzed, and it was found that the quality of the featured image is predicted by the first minima in the energy plots, demonstrated in the experimental analysis in Section 7.

The rest of the paper is organized as follows. In Section 2, we review the related work. Section 3 describes the research hypothesis. Section 4 presents a brief overview of Paillier encryption. Section 5 describes the system and the threat model of the architecture, details of the proposed PP-JPEG, and the solution. The security analysis is performed in Section 6. The performance of the proposed approach is outlined in Section 7. And the summary of the conclusion of the results is in Section 8. Furthermore, Section 9 concludes the work and discusses the future scope of the research.

## 2. Related Work

Various techniques have been studied in the field of detecting image forgery. Here, we provide a concise overview of some of these approaches.

Dospinescu et al. [7] propose fingerprint recognition; its significance lies in its role as a distinct identifier. This article covers its applications, evolution, and image pre-processing. An Android app called BioFinger, utilizing the SourceAFIS library, is introduced for fingerprint recognition using mobile device cameras, with results and future directions presented.

Amerini et al. [8] propose a step forward in this direction by analyzing how a single or double JPEG compression can be revealed and localized using convolutional neural networks (CNNs). Zhou et al. [9] propose a two-stream Faster R-CNN network for tampering detection in manipulated images. This method combines RGB and noise features, outperforming others on standard datasets and remaining robust to resizing and compression.

Ting et al. [10] propose a data-embedding method for the JPEG XT format, utilizing coefficient count variations between base and residual layers. Swapping MCUs between these layers embeds data while maintaining image quality or introducing distortion. Experiments were conducted to evaluate the effectiveness of the method.

Diallo et al. [11] propose a framework for detecting image forgery, which considers various transformations, such as compression and resizing. The framework is based on a convolutional neural network and considers the image quality for the application. Doegar et al. [12] address privacy and tampering issues in medical imaging and bioinformatics caused by cloud computing. Image-tampering detection is proposed using a deep learning architecture to identify tampered components efficiently. The proposed approach is evaluated on the MICC-F220 dataset using k-fold cross validation.

Rani et al. [6] propose a framework in detecting image manipulations like copy–move and splicing. It introduces a new pixel-based forgery detection framework using enhanced SURF and template matching. Evaluation on CASIA dataset shows 97% accuracy with enhanced SURF and 100% with template matching.

Doan et al. [13] introduce a novel method for detecting image manipulation in JPEG natural images using a signal-dependent noise model. It employs two fingerprints within a hypothesis-testing framework, supported by generalized likelihood ratio tests (GLRTs). Various types of forgery, such as resampling and filtering, are addressed, with experiments on real and simulated images demonstrating its effectiveness.

Ghaffarian et al. [14] explore the integration of attention mechanisms with deep learning in computer vision and remote sensing image processing. Attention mechanisms enhance deep learning performance. The research reviews 270 papers, finding that incorporating attention mechanisms consistently improves accuracy across tasks like image classification, object detection, and change detection in remote sensing.

Lu et al. [15] introduce an interpretable image forensics approach to counter image tampering, combining suspicious tampered region detection (STRD) and cooperative game modules. The STRD module detects tampering types, including small regions, in complex scenes. The cooperative game module employs the Shapley interaction index to measure information gained from pixels, focusing on the low-order effects of tampering. This approach showcases strong interpretability and outperforms other methods in experiments. Singh et al. [16] propose a framework that can detect image forgeries. The framework identifies the forged portion of the image, called the ghost image. It has a compression quality different from that of the cover image.

Rahmati et al. [17] propose a new method for detecting double JPEG compression in images using a convolutional auto-encoder and convolutional neural network. The method outperforms previous algorithms on standard datasets and is robust to perturbations in JPEG compression quality factors. Results are based on small-sized image patches.

Yu et al. [18] propose NOStyle, a noise-optimized stacked StyleGAN2, for secure, high-quality image synthesis. It works through two stages: the first generates a benchmark image, while the second adapts stochastic variation using a noise-secure optimization network. This yields secure images suitable for data hiding and maintaining quality. Experimental results show success and reveal a trade-off between image security and fidelity.

Kumar et al. [19] propose a method for detecting copy–move forgery in images using a reduced feature-based algorithm. It uses stationary wavelet transform to obtain the low approximation band of the subject image and then extracts significant features using block-based DCT and SVD. The approach only extracts three feature vectors to reduce the computational overhead but still achieves the precise detection of forged areas and is robust against post-processing attacks.

Our proposed image tamper detection scheme is highly robust and efficient, capable of detecting tampering in encrypted images with all possible combinations of JPEG quality for both dummy and featured images. By operating in an encrypted domain, our scheme ensures the privacy and security of the images while providing reliable tamper detection.

## 3. Research Hypothesis

Our work proposes a framework to detect image forgery in an encrypted domain. Earlier studies have focused on image forgery detection techniques that work in the plain-text domain. However, since we are working in an encrypted domain, we examine whether any difference exists between the pixel intensities of tampered and untampered images in the encrypted domain.

The existence of differences in pixel intensities between tampered and untampered images enables a framework to detect image forgery in the encrypted domain. We examine if the mean pixel intensities of tampered and untampered images in the encrypted domain are different. Based on this, we have the following hypotheses:

**H${}_{0}$:***The mean pixel intensities of tampered and untampered images are the same*.

**H${}_{a}$:***The mean pixel intensities of tampered and untampered images are different*.

Based on this, we perform a significance test, wherein we take samples of encrypted tampered and untampered images of different qualities and perform a *t*-test. Since the *p*-value $<1.525\times 10^{-19}$, we can reject the null hypothesis and accept the alternate hypothesis.

## 4. Preliminaries

### 4.1. Paillier Encryption

The Paillier cryptosystem, created and named after Pascal Paillier in 1999, is an asymmetric cryptographic algorithm used for public key cryptography with homomorphic properties [20].

#### 4.1.1. Key Generation

Choose two large prime numbers *p* and *q*, and a random value *g*, where *g* is a generator of $Z_{n}^{*}$. Set n=p·q and λ=lcm(p−1,q−1). The public key is (n,g), and the private key is $\mu =L{\left(g^{\lambda }\;\mathrm{mod}\;n^{2}\right)}^{-1}\;\mathrm{mod}\;n$, where L(x)=(x−1)/n.

#### 4.1.2. Encryption

To encrypt message *m*, choose random *r* such that 0≤r<n, and compute $c=g^{m}\cdot r^{n}\;\mathrm{mod}\;n^{2}$. *c* is the encrypted message.

#### 4.1.3. Decryption

To decrypt message *c*, compute $m=L(c^{\lambda }\;\mathrm{mod}\;n^{2})\cdot \mu \;\mathrm{mod}\;n$.

#### 4.1.4. Homomorphic Properties

Paillier encryption supports additive homomorphism, which means that given two encrypted messages $c_{1}$ and $c_{2}$ that represent messages $m_{1}$ and $m_{2}$, respectively, we can compute an encrypted message $c_{3}$ that represents the sum of $m_{1}$ and $m_{2}$, without decrypting any of the values.

This is done by simply multiplying $c_{1}$ and $c_{2}$ together, modulo $n^{2}$. That is,

$$c_{3}=c_{1}\cdot c_{2}\;\mathrm{mod}\;n^{2}$$

When we decrypt $c_{3}$ using the private key, we obtain the sum of $m_{1}$ and $m_{2}$ modulo *n*:

$$m_{1}+m_{2}\equiv \mathrm{Dec}\left(c_{1}\cdot c_{2}\;\mathrm{mod}\;n^{2}\right)\;\left(\mathrm{mod}\;n\right)$$

We can also compute a scalar multiplication of an encrypted message $c_{1}$ with a plaintext value *a* by raising $c_{1}$ to the power of *a*, modulo $n^{2}$:

$$c_{2}=c_{1}^{a}\;\mathrm{mod}\;n^{2}$$

When we decrypt $c_{2}$, we obtain the plaintext value $m_{2}$ that is equal to $a\cdot m_{1}$ modulo *n*:

$$m_{2}\equiv a\cdot m_{1}\;\left(\mathrm{mod}\;n\right)$$

These homomorphic properties make Paillier encryption a useful tool for secure computation on encrypted data, as it allows for the manipulation of data without the need to decrypt them first.

## 5. The Proposed Framework

**Figure 1:**
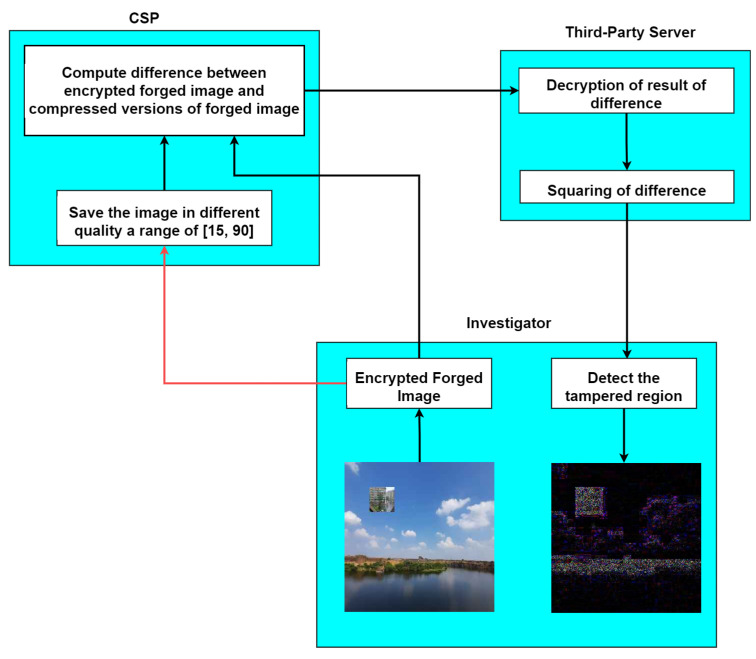
An overview of the proposed methodology.

Our proposed framework consists of three entities: the system model, the threat model, and the proposed methodology. These entities are shown in Figure 1.

### 5.1. System Model and Threat Model

The system model defines the structure of the system being analyzed, while the threat model identifies potential security threats. The proposed methodology outlines the steps used to assess and mitigate these threats within the system. This comprehensive framework provides an effective approach to addressing security concerns:

- **Investigator**: As an investigator, the role is to identify the forged region in an encrypted image without compromising the confidentiality of the image content. The forged images, denoted as $I{\left(x,y\right)}_{F}$, are created by copying and pasting a portion of an image onto another region within the same image and then saving the resulting image at different JPEG qualities [21]. The investigator performs this task by outsourcing the encrypted forged image to a cloud service provider (CSP), who is assumed to be honest but curious.
  The image is encrypted using Paillier encryption, a public-key encryption scheme that supports homomorphic operations to maintain the confidentiality of the image. The CSP follows the proposed protocol for detecting forged regions but may also be interested in learning about the content of the image. However, since Paillier encryption is semantically secure, the encryption scheme reveals no information about the underlying image content. This ensures the privacy of the original image while allowing the identification of any tampered regions. It is assumed that the investigator is a reliable and trusted entity.
- **Cloud service provider (CSP)**: The cloud service provider is an entity that resaves the encrypted forged image at different JPEG qualities and computes the difference between the encrypted forged image with the resaved image of different qualities. This ensures the confidentiality of the original image content throughout the process. Once the CSP has computed the encrypted differences, they can be outsourced to a third-party server for further analysis. We consider the communication channel to be insecure between the investigator and CSP, implying that the CSP is deemed to be an honest yet curious entity.
- **Third-party server**: As a third-party server, the primary responsibility is to assist the investigator in detecting forged regions in an encrypted image. This involves decrypting the encrypted difference between the resaved image received from the CSP.
  Once decrypted, the difference must be squared to amplify it before being sent back to the investigator. His role is to ensure that the decryption process is performed securely and that the squared difference is sent back to the investigator without any loss of data or privacy concerns. The third-party server is assumed to be a trusted entity.

### 5.2. Proposed Methodology

The proposed methodology presents a framework for verifying the integrity of an image in an encrypted domain as shown in Figure 1. This approach uses the characteristics of JPEG compression to find areas in images that have been altered, all while keeping the original image content confidential. The steps below provide a detailed explanation of the process:

- **Investigator**
  To verify the integrity of a potentially tampered image, the investigator follows these steps:
  **Step 1:** An image is provided to the investigator so that its integrity can be checked. For example, suppose that a section measuring 30 by 30 pixels from an image, which was saved with JPEG quality 50 and is referred to as a “dummy image”, is copied onto another image, known as the “featured image”, which was initially saved at quality 80. In this scenario, the resulting image is determined to be a manipulated image.
  **Step 2:** The forged image is encrypted using the public key of Paillier encryption [22] $E(I{\left(x,y\right)}_{F})$. It is sent to the CSP.
- **CSP**
  **Step 3:** The integrity of the forged image $E(I{\left(x,y\right)}_{F})$ is checked by resaving at different JPEG qualities $E(I{\left(x,y\right)}_{Fq})$, where *q* is the quality of the image. We performed this for a range of [15, 90] with a step size of 5.
  **Step 4:** To compute the difference image E(S) in the encrypted domain, the operations involved are addition and scalar multiplication, which are supported by Paillier encryption. The sum of two plaintexts $I_{1}$ and $I_{2}$ is equivalent to the decrypted product of corresponding ciphertexts $E(I_{1})$ and $E(I_{2})$. The product of a scalar with a plaintext $I_{1}$ is equivalent to the decrypted exponentiation of the corresponding ciphertext $E(I_{1})$ with the scalar. In the plaintext domain, the difference image *S* is obtained by computing between the plaintext forged image *I* and the resaved image $I^{q}$ as shown in Equation (1). In the encrypted domain, the difference image E(S) is obtained by computing between the encrypted forged image E(I) and the resaved image $E(I^{q})$ as shown in Equation (2):
  **Difference in plaintext domain:**
  (1) $$S\left(x,y\right)=[{I{\left(x,y\right)}_{Fi}-I{\left(x,y\right)}_{Fi}^{q}]}_{i=1,2,3}$$
  **Difference in encrypted domain:**
  (2) $$E\left(S\left(x,y\right)\right)=[{E\left(I{\left(x,y\right)}_{Fi}\right)\times {\left(E\left(I{\left(x,y\right)}_{Fi}^{q}\right)\right)}^{-1}]}_{i=1,2,3}$$
  where $E(I{\left(x,y\right)}_{Fi})$ and $E(I{\left(x,y\right)}_{Fi}^{q})$ represent the pixel value at (x,y) co-ordinates of the $i^{th}$ color channel (red (R), green (G), and blue (B) channels) of the forged image and resaved forged image, respectively.
- **Third-Party Server**
  **Step 5:** After receiving the encrypted difference E(S) as shown in Equation (2) from the CSP, the third-party server decrypts it using the private key of the Paillier encryption scheme. As squaring cannot be performed in the encrypted domain, the decrypted difference is squared to amplify the difference as follows:
  (3) $$D\left(S\left(x,y\right)\right)={\left(Dec\left[E\left(S\left(x,y\right)\right)\right]\right)}_{i=1,2,3}^{2}$$
  Here, Dec represents the decryption operation and D(S(x,y)), referred to in Equation (3), represents the squared difference value at the (x,y) co-ordinate of the *i*-th color channel.
- **Investigator**
  **Step 6:** The decrypted and squared difference image D(S) is received from the third-party server and converted to RGB format. This step allows the forged regions to be visualized more clearly, and the tampered portions of the image can be identified with greater detail in the experimental analysis in Section 7.

## 6. Security Analysis

Paillier encryption is a form of homomorphic encryption that is semantically secure and probabilistically correct, and it can be useful in privacy-preserving protocols [20].

**Theorem** **1.** *If the Paillier encryption is semantically secure, PP-JPEG is also semantically secure.*

**Proof.**  The proof of semantic security in Paillier encryption is based on the decisional composite residuosity assumption (DCR). DCR states that given a composite number *n* and a random number *a*, it is computationally infeasible to determine whether gcd(a,n)=1 or $a^{\lambda (n)}=1\;\mathrm{mod}\;n^{2}$, where λ is the Carmichael function.

To prove the semantic security of Paillier encryption, we show that given any two plaintexts $m_{1}$ and $m_{2}$, the difference between their corresponding ciphertexts $C_{1}$ and $C_{2}$ is statistically indistinguishable from a uniformly random value in $Z_{n^{2}}$.

Let us consider the ciphertexts

$$C_{1}=\left(g^{m_{1}}\cdot r^{n}\right)\;\mathrm{mod}\;n^{2}$$

$$C_{2}=\left(g^{m_{2}}\cdot r^{n}\right)\;\mathrm{mod}\;n^{2}$$

where *g* is a generator of the group, *n* is the public key modulus, *r* is a random value chosen during encryption, and mod denotes the modulus operation.

The difference between the two ciphertexts can be expressed as

$$\begin{array}{c} \left(C_{1}\cdot C_{2}^{-1}\right)\;\mathrm{mod}\;n^{2}\\ \;=\left[\left(g^{m_{1}}\cdot r^{n}\right)\cdot \left(g^{-m_{2}}\cdot r^{-n}\right)\right]\;\mathrm{mod}\;n^{2}\\ \;=g^{m_{1}-m_{2}}\;\mathrm{mod}\;n^{2} \end{array}$$

Now, let us consider a hypothetical attacker who is trying to distinguish between two ciphertexts $C_{1}$ and $C_{2}$ without knowing the corresponding plaintexts.

The attacker can compute the value $g^{m_{1}-m_{2}}\;\mathrm{mod}\;n^{2}$ based on the ciphertexts. However, under the DCR assumption, this value is computationally indistinguishable from a uniformly random value in $Z_{n^{2}}$, unless the factorization of *n* is known. Therefore, the attacker cannot gain any information about the difference between the plaintexts $m_{1}$ and $m_{2}$ solely based on the ciphertexts. This demonstrates the semantic security of the Paillier encryption scheme. In conclusion, the Paillier encryption scheme is semantically secure under the decisional composite residuosity assumption (DCR). Without knowledge of the private key, an attacker cannot determine the corresponding plaintext from the ciphertext, as the difference between ciphertexts reveals no information about the plaintexts. □

**Theorem** **2.** *If the Paillier encryption is probabilistically correct, PP-JPEG is also probabilistically correct.*

**Proof.**  Let *m* be the plaintext message, *r* be a random value, *g* be a generator of the group, and *n* be the public key modulus.

The ciphertext *C* is computed as

$$C=\left(g^{m}\cdot r^{n}\right)\;\mathrm{mod}\;n^{2}$$

where · denotes multiplication, mod denotes the modulus operation, and all operations are performed within the group.

This formula ensures the probabilistic correctness of Paillier encryption, as the random value *r* is added to the plaintext before encryption and removed during decryption. The resulting ciphertext *C* is a representation of the encrypted message that cannot be easily reversed to obtain the original plaintext without knowing the private key. □

## 7. Experimental Analysis

Our experiments were conducted using Intel(R) Xeon(R) Silver 4214R CPU @ 2.40 GHz. We considered various scenarios to test the effectiveness of our proposed method. We tested the method on different combinations of dummy and featured images, such as high-quality dummy image with low-quality featured image, low-quality dummy image with high-quality featured image, and dummy and featured images with the same quality. Additionally, we examined the method’s ability to detect even small forgeries by testing it on dummy images of sizes ranging from 50×50 to 10×10 pixels. The results for each of these scenarios are presented in the following section:

### Result Analysis

**Figure 2:**
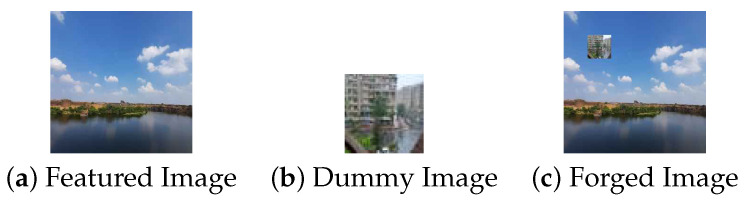
Forged image, where the forgery portion is 50×50.

**Figure 3:**
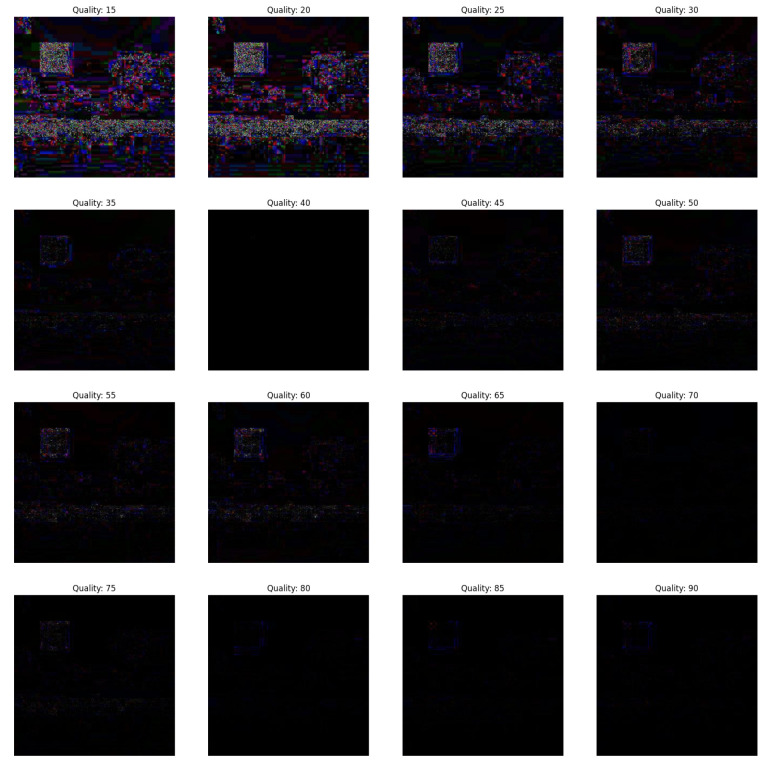
The forged portion is 50×50, where the featured image quality is 40 and dummy image quality is 70.

**Figure 4:**
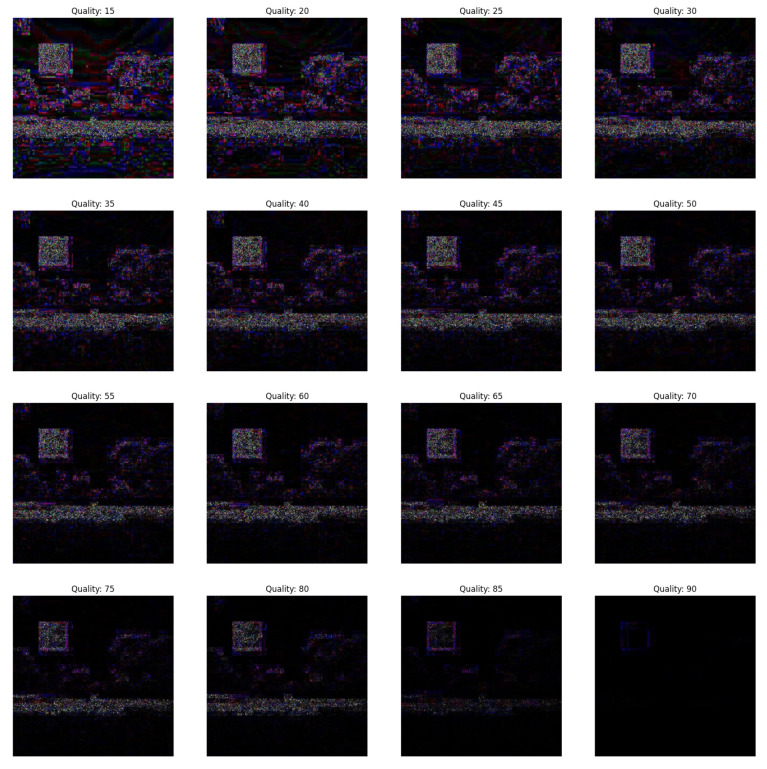
The forged portion is 50×50, where the featured image quality is 90 and dummy image quality is 70.

**Figure 5:**
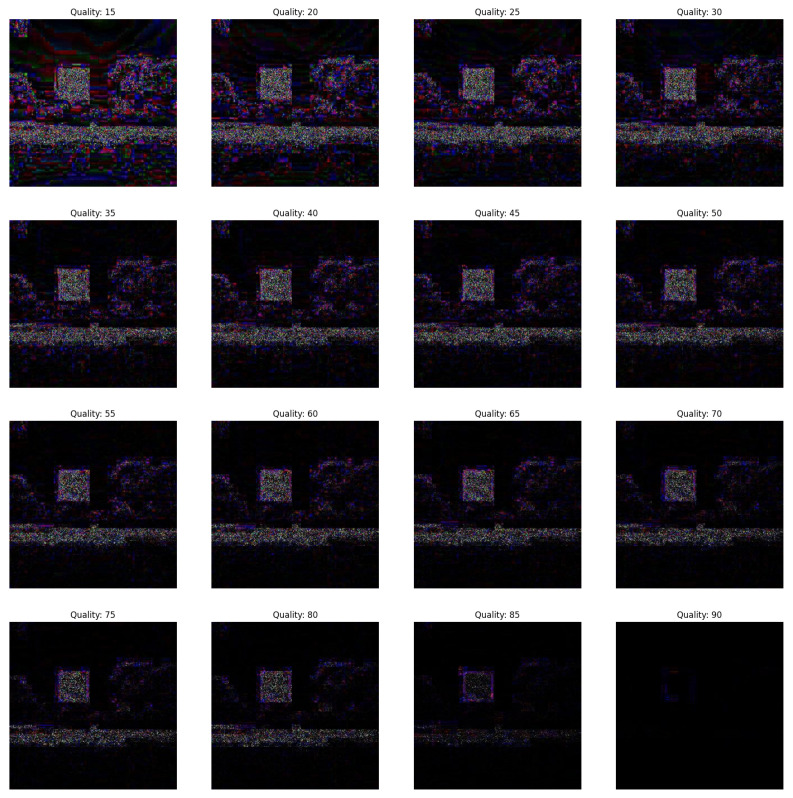
The forged portion is 50×50 where the featured image quality is 90 and dummy image quality is 90.

**Figure 6:**
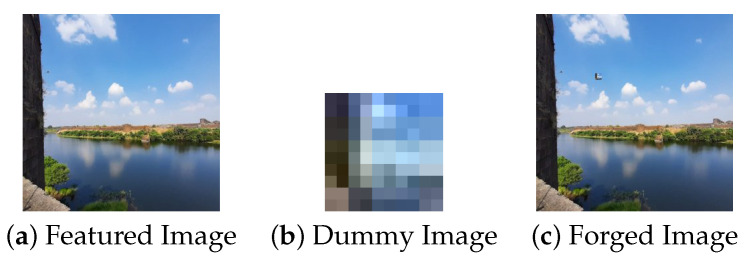
Forged image, where the forgery portion is 10×10.

**Figure 7:**
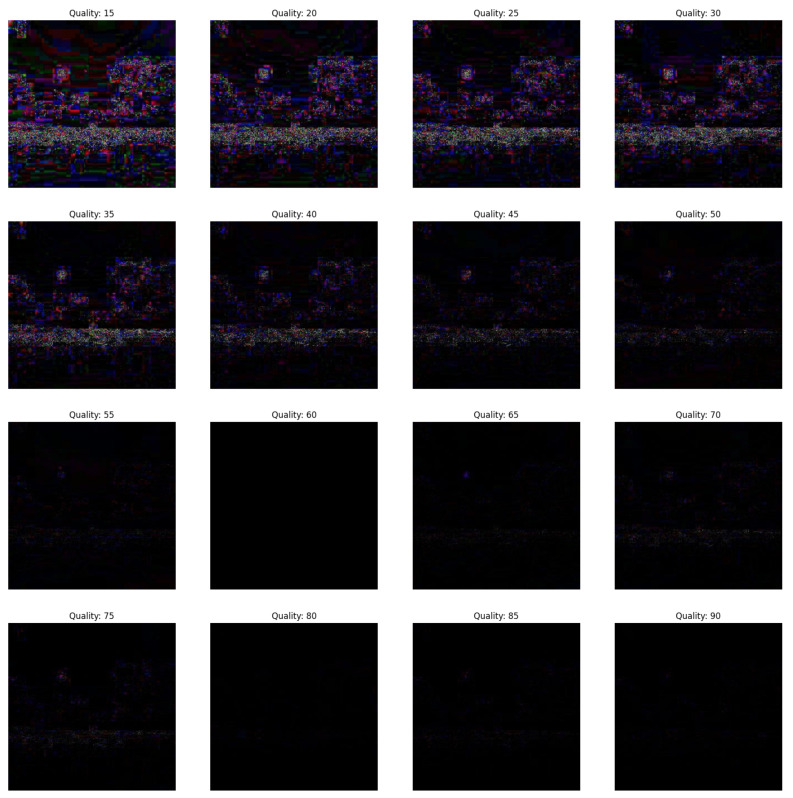
The forged portion 10×10, where the featured image quality is 60 and dummy image quality is 85.

**Figure 8:**
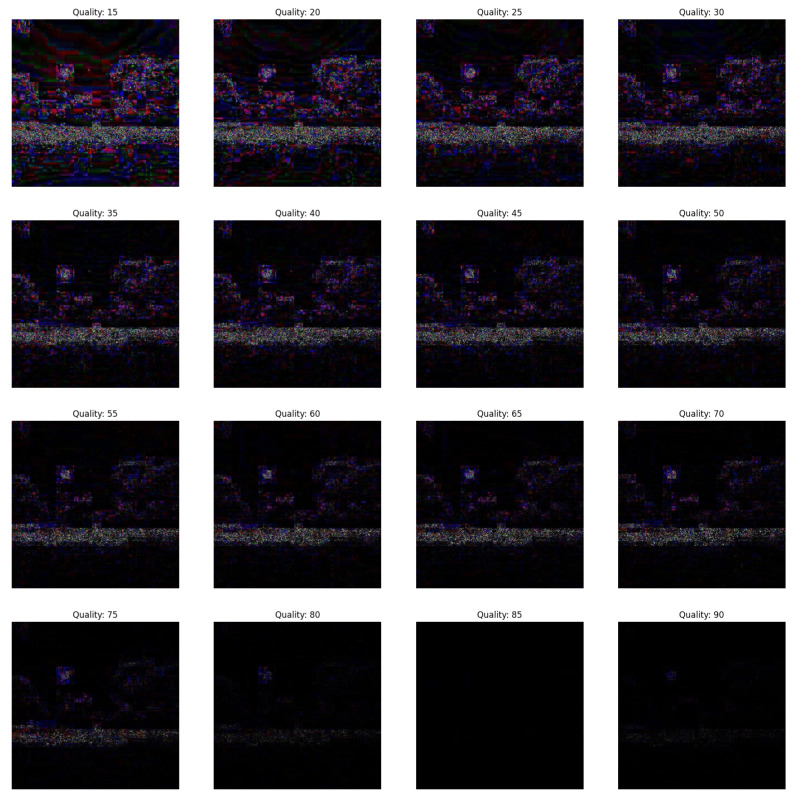
The forged portion 10×10, where the featured image quality is 85 and dummy image quality is 60.

**Figure 9:**
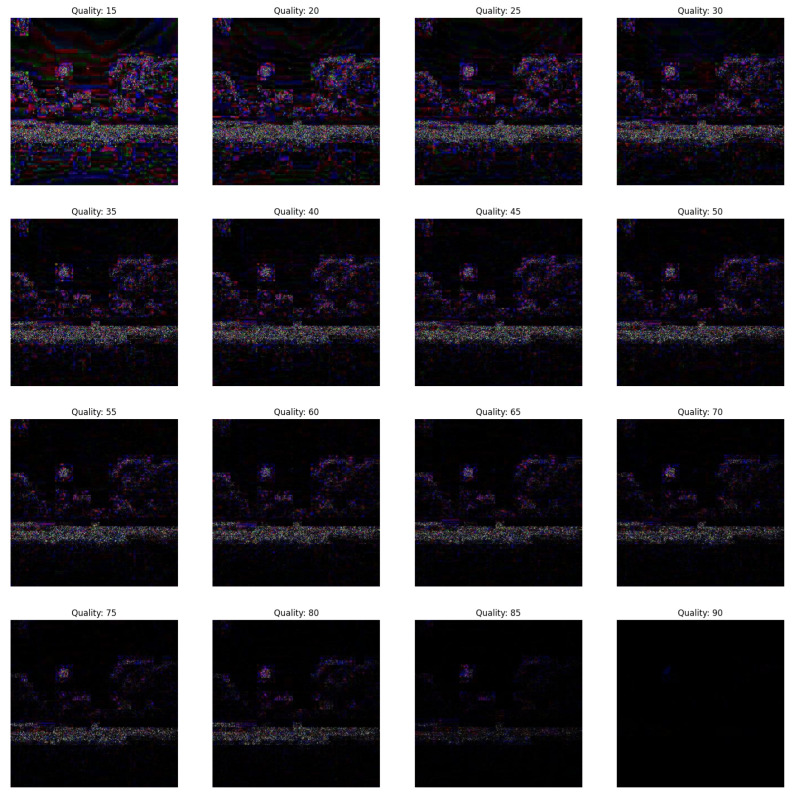
The forged portion is 10×10, where the featured image quality is 90 and dummy image quality is 90.

1. In the first scenario, there are three conditions where the forgery portion size in all the conditions is 50×50 as shown in Figure 2. The first condition involves a dummy image with higher quality than the featured image, while the second condition involves the featured image having higher quality than the dummy image, and the third condition involves the featured image having equal quality to the dummy image.
  (a) In Figure 3, the featured image quality is 40 and dummy image quality is 70 of size 50×50 inserted at coordinate (50, 50).
  (b) In Figure 4, the featured image quality is 90 and dummy image quality is 70 of size 50×50 inserted at coordinate (50, 50).
  (c) In Figure 5, the featured image quality is 90 and dummy image quality is 90 of size 50×50 inserted at coordinate (90, 90).
2. In the last scenario, there are three conditions, where the forgery portion size in all conditions is 10×10 as shown in Figure 6. The first condition involves a dummy image with higher quality than the featured image, while the second condition involves the featured image having higher quality than the dummy image, and the third condition involves the featured image having equal quality to the dummy image.
  (a) In Figure 7, the featured image quality is 60 and dummy image quality is 85 of size 10×10 inserted at coordinate (90, 90).
  (b) In Figure 8, the featured image quality is 85 and dummy image quality is 60 of size 10×10 inserted at coordinate (90, 90).
  (c) In Figure 9, the featured image quality is 90 and dummy image quality is 90 of size 10×10 inserted at coordinate (90, 90).

Our experiments show that the proposed approach effectively detects the forgery in the encrypted domain. In simpler terms, our approach works well for all JPEG quality levels and all potential combinations of dummy and featured encrypted images.

**Figure 10:**
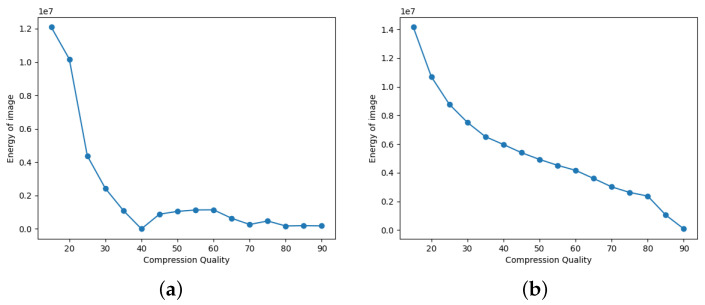
Energy graph for the forgery portion 50×50. (**a**) Energy graph where featured image quality is 40 with reference to Figure 3; (**b**) energy graph where featured image quality is 90 with reference to Figure 4.

**Figure 11:**
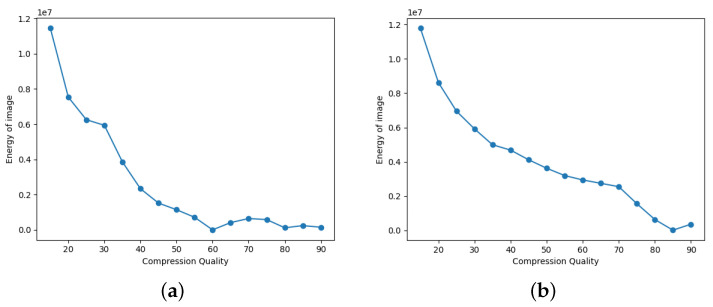
Energy graph for the forgery portion 10×10. (**a**) Energy graph where featured image quality is 60 with reference to Figure 7; (**b**) energy graph where featured image quality is 85 with reference to Figure 8.

Additionally, we examine another experimental scenario based on the difference between the forged and resaved versions of the image and the difference is calculated as shown in Equation (2). P(x,y) is the energy of the image as shown in Equation (4):

(4) $$P\left(x,y\right)=\sum_{d=1}^{dim}\sum_{x=1}^{rows}\sum_{y=1}^{col}S\left(x,y,d\right)$$

where P(x,y) represents the sum of the amplified pixel values of the decrypted difference as shown in Equation (3). The quality of the featured image is indicated by the first minima in graphs of “energy of image” against its “compression quality”. In Figure 10 and Figure 11, the first minima occur at a compression quality that corresponds to the quality of the featured image; along with that, the minute forgeries can be identified using our proposed scheme.

## 8. Discussion

In Section 3, we validated through the *t*-test that the *p*-value $<1.525\times 10^{-19}$, so we can reject the null hypothesis and accept the alternate hypothesis. Thus, there exists a difference in the pixel intensities of tampered and untampered images in the encrypted domain, and a framework can work to detect image forgery in this domain. Our method effectively detected forged regions across various JPEG quality levels and image combinations in the encrypted domain, regardless of image quality and composition. In contrast to Singh et al. [16], who focus on JPEG image forensic techniques based on compression quality differences, our work addresses privacy concerns alongside forgery detection. We propose a privacy-preserving framework that encrypts images pre-processing, ensuring confidentiality. Highlighting localization and JPEG quality identification, our approach uniquely emphasizes confidentiality and provides a valuable solution for image forgery detection in sensitive contexts. Our proposed work presents a more comprehensive and advanced solution than Rani et al. [6]. While Rani et al. [6] address limitations in detecting specific manipulations, the proposed work tackles the broader challenges of privacy and authenticity in image processing. Our privacy-preserving framework encrypts images before processing, ensuring confidentiality, a dimension not covered by Rani et al. [6]. This approach’s sophistication lies in employing compression quality analysis within the encrypted domain to identify forgeries. Moreover, our work has the ability to pinpoint tampered regions, even in small pixel blocks. Doan et al. [13] focus on signal-dependent noise models and hypothesis testing theory to detect manipulation using noise patterns. Our proposed work prioritizes privacy with encryption and compression analysis. It connects manipulation, privacy, and real-world contexts like law enforcement. It highlights data privacy importance and provides evidence of effectiveness, including small pixel block detection (10×10). The proposed framework can be used in various tampering detection scenarios within the encrypted domain. It can effectively detect tampering in multiple contexts, such as fingerprint images by ensuring the integrity of biometric data, legal image evidence by verifying the authenticity of legal evidence, remote sensing imagery by detecting any fraudulent changes in satellite or aerial images used for remote-sensing applications, document images identifying any falsification or alterations in digital documents, preserving the integrity of textual information, etc. The framework is valuable in safeguarding data integrity across multiple domains. However, one limitation is observed when the feature image quality is equal to that of the dummy image, which could be further improved in future research.

## 9. Conclusions and Future Work

In this paper, we described a privacy-preserving framework for detecting tampering in JPEG images in the encrypted domain. In our experiments, we found that combining two different JPEG images of different quality is quite likely to lead to forgery detection. The energy graph can be used to determine the quality of the featured image. As shown in the energy graph, the quality of the featured image is the first minima. However, the chances of forgery detection are quite low when the JPEG quality is the same. No matter whether the images were captured from the same camera device or not, this holds true. In future work, we can extend to other image formats, as our paper focuses on JPEG images. Further, we intend to extend our work by using our architecture in a multi-CPU setup. This configuration would enable us to leverage multiprocessing techniques for the encryption of images. Furthermore, it would be interesting to explore how the proposed method could be adapted in a multiprocessing setup and to extend our approach with other image formats, such as PNG, BMP, or TIFF.

## Data Availability

Not applicable.
